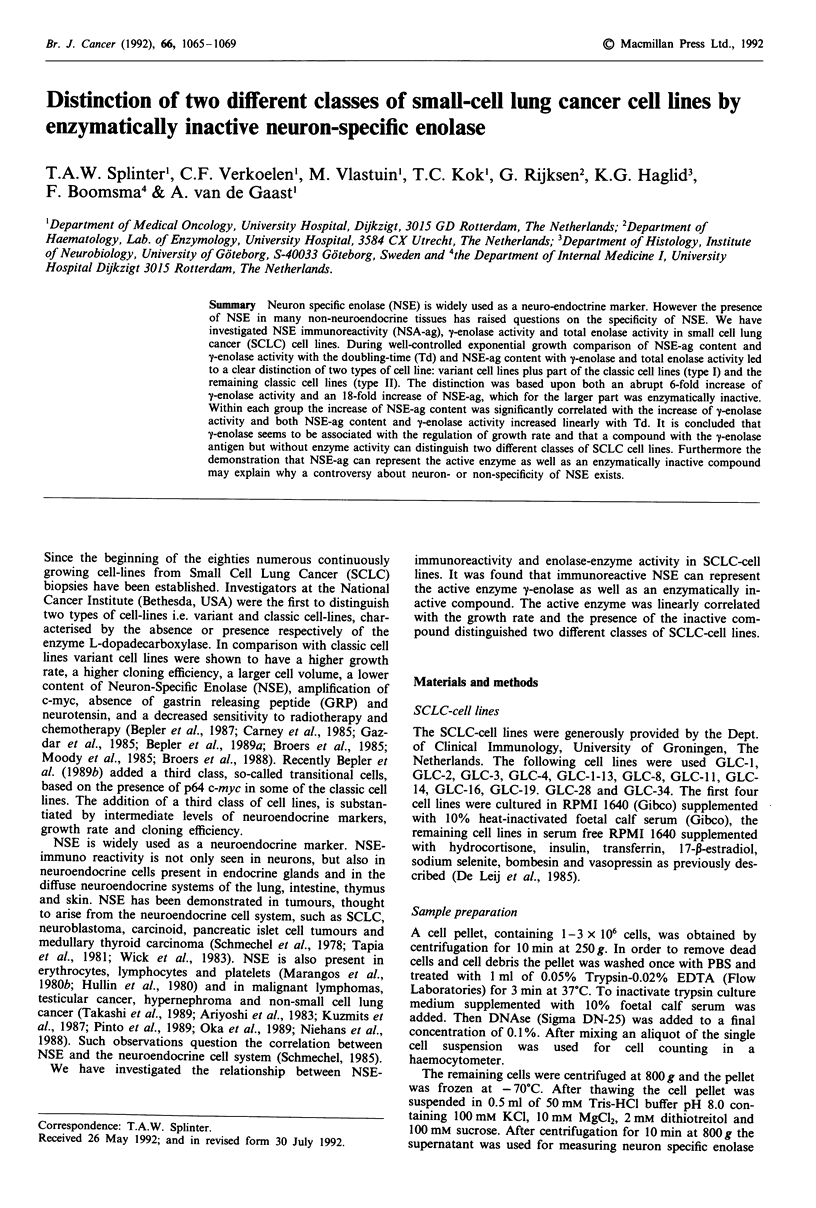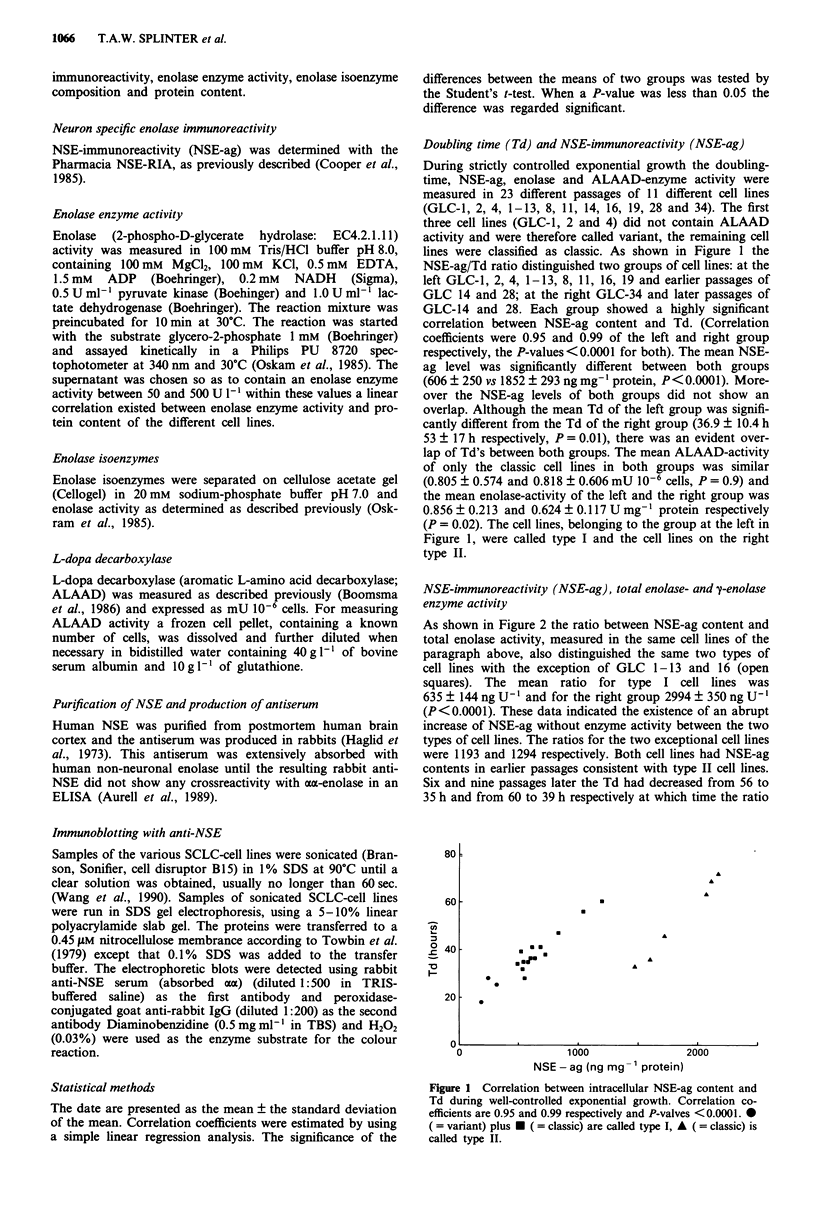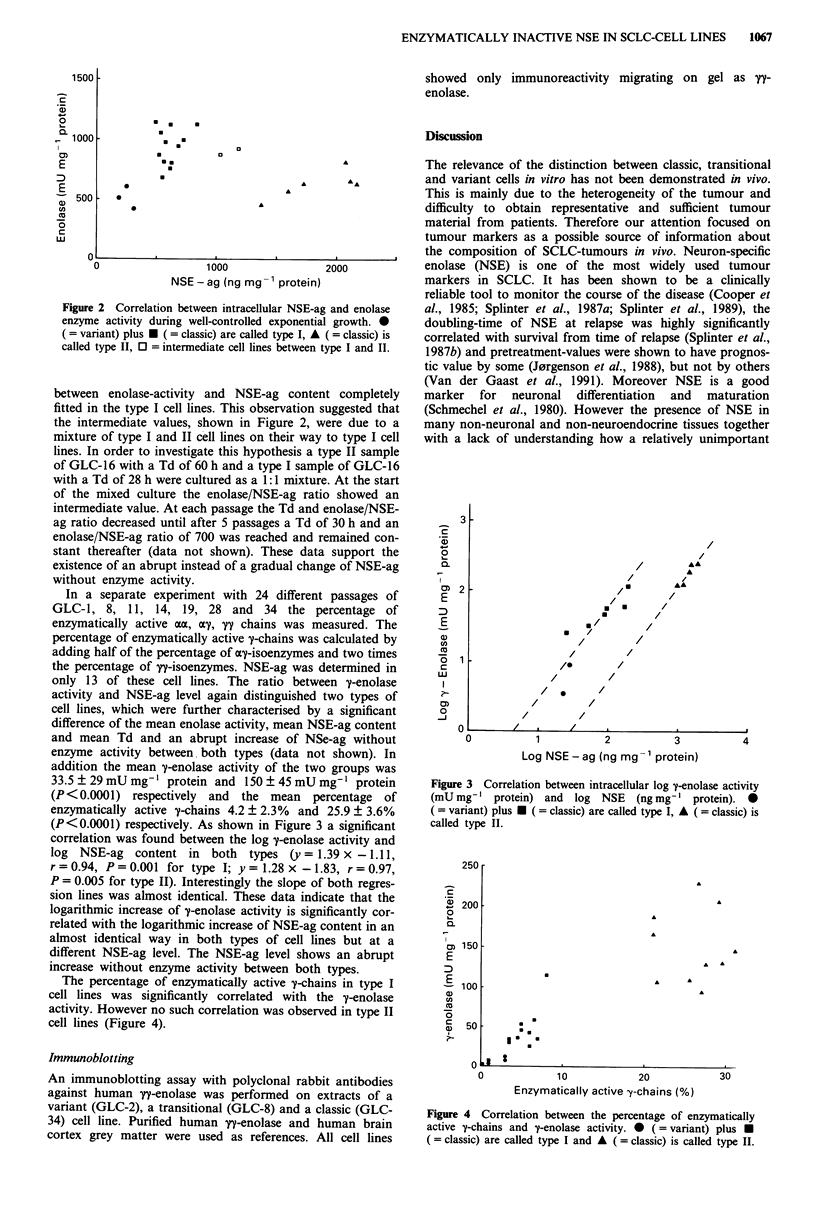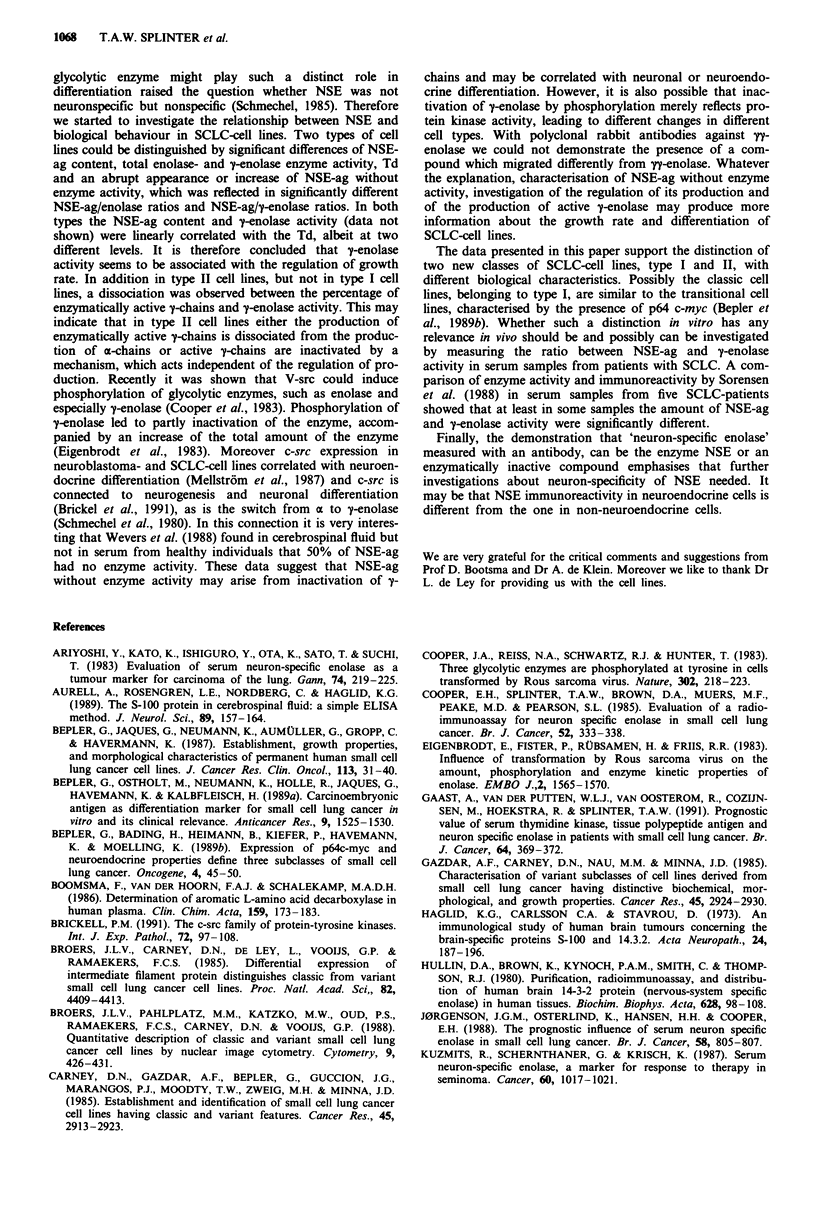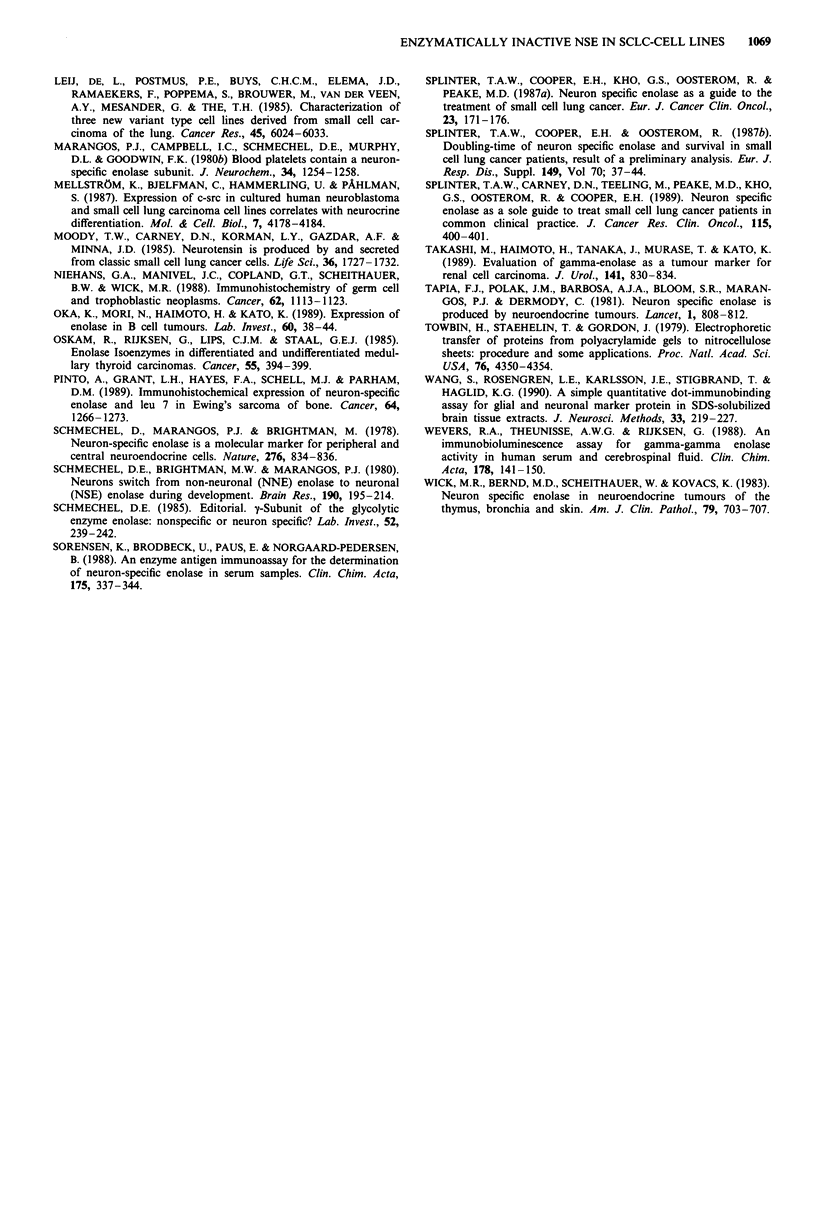# Distinction of two different classes of small-cell lung cancer cell lines by enzymatically inactive neuron-specific enolase.

**DOI:** 10.1038/bjc.1992.411

**Published:** 1992-12

**Authors:** T. A. Splinter, C. F. Verkoelen, M. Vlastuin, T. C. Kok, G. Rijksen, K. G. Haglid, F. Boomsma, A. van de Gaast

**Affiliations:** Department of Medical Oncology, University Hospital, Rotterdam, The Netherlands.

## Abstract

Neuron specific enolase (NSE) is widely used as a neuro-endocrine marker. However the presence of NSE in many non-neuroendocrine tissues has raised questions on the specificity of NSE. We have investigated NSE immunoreactivity (NSA-ag), gamma-enolase activity and total enolase activity in small cell lung cancer (SCLC) cell lines. During well-controlled exponential growth comparison of NSE-ag content and gamma-enolase activity with the doubling-time (Td) and NSE-ag content with gamma-enolase and total enolase activity led to a clear distinction of two types of cell line: variant cell lines plus part of the classic cell lines (type I) and the remaining classic cell lines (type II). The distinction was based upon both an abrupt 6-fold increase of gamma-enolase activity and an 18-fold increase of NSE-ag, which for the larger part was enzymatically inactive. Within each group the increase of NSE-ag content was significantly correlated with the increase of gamma-enolase activity and both NSE-ag content and gamma-enolase activity increased linearly with Td. It is concluded that gamma-enolase seems to be associated with the regulation of growth rate and that a compound with the gamma-enolase antigen but without enzyme activity can distinguish two different classes of SCLC cell lines. Furthermore the demonstration that NSE-ag can represent the active enzyme as well as an enzymatically inactive compound may explain why a controversy about neuron- or non-specificity of NSE exists.


Br. J. Cancer (1992), 66, 1065-1069 © Macmillan Press Ltd., 1992

### Distinction of two different classes of small-cell lung cancer cell lines by enzymatically inactive neuron-specific enolase

T.A.W. Splinter[1], C.F. Verkoelen[1], M. Vlastuin[1], T.C. Kok[1], G. Rijksen[2], K.G. Haglid[3], F. Boomsma[4] & A. van de Gaast[1]

[1]*Department of Medical Oncology, University Hospital, Dijkzigt, 3015 GD Rotterdam, The Netherlands;* [2]*Department of Haematology, Lab. of Enzymology, University Hospital, 3584 CX Utrecht, The Netherlands;* [3]*Department of Histology, Institute of Neurobiology, University of Göteborg, S-40033 Göteborg, Sweden and* [4]*the Department of Internal Medicine I, University Hospital Dijkzigt 3015 Rotterdam, The Netherlands.*

**Summary** Neuron specific enolase (NSE) is widely used as a neuro-endoctrine marker. However the presence of NSE in many non-neuroendocrine tissues has raised questions on the specificity of NSE. We have investigated NSE immunoreactivity (NSA-ag), γ-enolase activity and total enolase activity in small cell lung cancer (SCLC) cell lines. During well-controlled exponential growth comparison of NSE-ag content and γ-enolase activity with the doubling-time (Td) and NSE-ag content with γ-enolase and total enolase activity led to a clear distinction of two types of cell line: variant cell lines plus part of the classic cell lines (type I) and the remaining classic cell lines (type II). The distinction was based upon both an abrupt 6-fold increase of γ-enolase activity and an 18-fold increase of NSE-ag, which for the larger part was enzymatically inactive. Within each group the increase of NSE-ag content was significantly correlated with the increase of γ-enolase activity and both NSE-ag content and γ-enolase activity increased linearly with Td. It is concluded that γ-enolase seems to be associated with the regulation of growth rate and that a compound with the γ-enolase antigen but without enzyme activity can distinguish two different classes of SCLC cell lines. Furthermore the demonstration that NSE-ag can represent the active enzyme as well as an enzymatically inactive compound may explain why a controversy about neuron- or non-specificity of NSE exists.

Since the beginning of the eighties numerous continuously growing cell-lines from Small Cell Lung Cancer (SCLC) biopsies have been established. Investigators at the National Cancer Institute (Bethesda, USA) were the first to distinguish two types of cell-lines i.e. variant and classic cell-lines, characterised by the absence or presence respectively of the enzyme L-dopadecarboxylase. In comparison with classic cell lines variant cell lines were shown to have a higher growth rate, a higher cloning efficiency, a larger cell volume, a lower content of Neuron-Specific Enolase (NSE), amplification of c-myc, absence of gastrin releasing peptide (GRP) and neurotensin, and a decreased sensitivity to radiotherapy and chemotherapy (Bepler *et al.*, 1987; Carney *et al.*, 1985; Gazdar *et al.*, 1985; Bepler *et al.*, 1989*a*; Broers *et al.*, 1985; Moody *et al.*, 1985; Broers *et al.*, 1988). Recently Bepler *et al.* (1989*b*) added a third class, so-called transitional cells, based on the presence of p64 c-*myc* in some of the classic cell lines. The addition of a third class of cell lines, is substantiated by intermediate levels of neuroendocrine markers, growth rate and cloning efficiency.

NSE is widely used as a neuroendocrine marker. NSE-immuno reactivity is not only seen in neurons, but also in neuroendocrine cells present in endocrine glands and in the diffuse neuroendocrine systems of the lung, intestine, thymus and skin. NSE has been demonstrated in tumours, thought to arise from the neuroendocrine cell system, such as SCLC, neuroblastoma, carcinoid, pancreatic islet cell tumours and medullary thyroid carcinoma (Schmechel *et al.*, 1978; Tapia *et al.*, 1981; Wick *et al.*, 1983). NSE is also present in erythrocytes, lymphocytes and platelets (Marangos *et al.*, 1980*b*; Hullin *et al.*, 1980) and in malignant lymphomas, testicular cancer, hypernephroma and non-small cell lung cancer (Takashi *et al.*, 1989; Ariyoshi *et al.*, 1983; Kuzmits *et al.*, 1987; Pinto *et al.*, 1989; Oka *et al.*, 1989; Niehans *et al.*, 1988). Such observations question the correlation between NSE and the neuroendocrine cell system (Schmechel, 1985).

We have investigated the relationship between NSE-immunoreactivity and enolase-enzyme activity in SCLC-cell lines. It was found that immunoreactive NSE can represent the active enzyme γ-enolase as well as an enzymatically inactive compound. The active enzyme was linearly correlated with the growth rate and the presence of the inactive compound distinguished two different classes of SCLC-cell lines.

### Materials and methods

### *SCLC-cell lines*

The SCLC-cell lines were generously provided by the Dept. of Clinical Immunology, University of Groningen, The Netherlands. The following cell lines were used GLC-1, GLC-2, GLC-3, GLC-4, GLC-1-13, GLC-8, GLC-11, GLC-14, GLC-16, GLC-19. GLC-28 and GLC-34. The first four cell lines were cultured in RPMI 1640 (Gibco) supplemented with 10% heat-inactivated foetal calf serum (Gibco), the remaining cell lines in serum free RPMI 1640 supplemented with hydrocortisone, insulin, transferrin, 17-β-estradiol, sodium selenite, bombesin and vasopressin as previously described (De Leij *et al.*, 1985).

### *Sample preparation*

A cell pellet, containing $1-3 \times 10^6$ cells, was obtained by centrifugation for 10 min at 250 *g*. In order to remove dead cells and cell debris the pellet was washed once with PBS and treated with 1 ml of 0.05% Trypsin-0.02% EDTA (Flow Laboratories) for 3 min at 37°C. To inactivate trypsin culture medium supplemented with 10% foetal calf serum was added. Then DNAse (Sigma DN-25) was added to a final concentration of 0.1%. After mixing an aliquot of the single cell suspension was used for cell counting in a haemocytometer.

The remaining cells were centrifuged at 800 *g* and the pellet was frozen at −70°C. After thawing the cell pellet was suspended in 0.5 ml of 50 mM Tris-HCl buffer pH 8.0 containing 100 mM KCl, 10 mM $MgCl_2$, 2 mM dithiotreitol and 100 mM sucrose. After centrifugation for 10 min at 800 *g* the supernatant was used for measuring neuron specific enolase

Correspondence: T.A.W. Splinter.

Received 26 May 1992; and in revised form 30 July 1992.

immunoreactivity, enolase enzyme activity, enolase isoenzyme composition and protein content.

*Neuron specific enolase immunoreactivity*

NSE-immunoreactivity (NSE-ag) was determined with the Pharmacia NSE-RIA, as previously described (Cooper *et al.*, 1985).

*Enolase enzyme activity*

Enolase (2-phospho-D-glycerate hydrolase: EC4.2.1.11) activity was measured in 100 mM Tris/HCl buffer pH 8.0, containing 100 mM $MgCl_2$, 100 mM KCl, 0.5 mM EDTA, 1.5 mM ADP (Boehringer), 0.2 mM NADH (Sigma), 0.5 U $ml^{-1}$ pyruvate kinase (Boehinger) and 1.0 U $ml^{-1}$ lactate dehydrogenase (Boehringer). The reaction mixture was preincubated for 10 min at 30°C. The reaction was started with the substrate glycero-2-phosphate 1 mM (Boehringer) and assayed kinetically in a Philips PU 8720 spectophotometer at 340 nm and 30°C (Oskam *et al.*, 1985). The supernatant was chosen so as to contain an enolase enzyme activity between 50 and 500 U $l^{-1}$ within these values a linear correlation existed between enolase enzyme activity and protein content of the different cell lines.

*Enolase isoenzymes*

Enolase isoenzymes were separated on cellulose acetate gel (Cellogel) in 20 mM sodium-phosphate buffer pH 7.0 and enolase activity as determined as described previously (Oskram *et al.*, 1985).

*L-dopa decarboxylase*

L-dopa decarboxylase (aromatic L-amino acid decarboxylase; ALAAD) was measured as described previously (Boomsma *et al.*, 1986) and expressed as mU $10^{-6}$ cells. For measuring ALAAD activity a frozen cell pellet, containing a known number of cells, was dissolved and further diluted when necessary in bidistilled water containing 40 g $l^{-1}$ of bovine serum albumin and 10 g $l^{-1}$ of glutathione.

*Purification of NSE and production of antiserum*

Human NSE was purified from postmortem human brain cortex and the antiserum was produced in rabbits (Haglid *et al.*, 1973). This antiserum was extensively absorbed with human non-neuronal enolase until the resulting rabbit anti-NSE did not show any crossreactivity with αα-enolase in an ELISA (Aurell *et al.*, 1989).

*Immunoblotting with anti-NSE*

Samples of the various SCLC-cell lines were sonicated (Branson, Sonifier, cell disruptor B15) in 1% SDS at 90°C until a clear solution was obtained, usually no longer than 60 sec. (Wang *et al.*, 1990). Samples of sonicated SCLC-cell lines were run in SDS gel electrophoresis, using a 5–10% linear polyacrylamide slab gel. The proteins were transferred to a 0.45 μM nitrocellulose membrance according to Towbin *et al.* (1979) except that 0.1% SDS was added to the transfer buffer. The electrophoretic blots were detected using rabbit anti-NSE serum (absorbed αα) (diluted 1:500 in TRIS-buffered saline) as the first antibody and peroxidase-conjugated goat anti-rabbit IgG (diluted 1:200) as the second antibody Diaminobenzidine (0.5 mg $ml^{-1}$ in TBS) and $H_2O_2$ (0.03%) were used as the enzyme substrate for the colour reaction.

*Statistical methods*

The date are presented as the mean ± the standard deviation of the mean. Correlation coefficients were estimated by using a simple linear regression analysis. The significance of the differences between the means of two groups was tested by the Student's *t*-test. When a *P*-value was less than 0.05 the difference was regarded significant.

*Doubling time (Td) and NSE-immunoreactivity (NSE-ag)*

During strictly controlled exponential growth the doubling-time, NSE-ag, enolase and ALAAD-enzyme activity were measured in 23 different passages of 11 different cell lines (GLC-1, 2, 4, 1–13, 8, 11, 14, 16, 19, 28 and 34). The first three cell lines (GLC-1, 2 and 4) did not contain ALAAD activity and were therefore called variant, the remaining cell lines were classified as classic. As shown in Figure 1 the NSE-ag/Td ratio distinguished two groups of cell lines: at the left GLC-1, 2, 4, 1–13, 8, 11, 16, 19 and earlier passages of GLC 14 and 28; at the right GLC-34 and later passages of GLC-14 and 28. Each group showed a highly significant correlation between NSE-ag content and Td. (Correlation coefficients were 0.95 and 0.99 of the left and right group respectively, the *P*-values $<0.0001$ for both). The mean NSE-ag level was significantly different between both groups (606 ± 250 *vs* 1852 ± 293 ng $mg^{-1}$ protein, $P<0.0001$). Moreover the NSE-ag levels of both groups did not show an overlap. Although the mean Td of the left group was significantly different from the Td of the right group (36.9 ± 10.4 h 53 ± 17 h respectively, $P = 0.01$), there was an evident overlap of Td's between both groups. The mean ALAAD-activity of only the classic cell lines in both groups was similar (0.805 ± 0.574 and 0.818 ± 0.606 mU $10^{-6}$ cells, $P = 0.9$) and the mean enolase-activity of the left and the right group was 0.856 ± 0.213 and 0.624 ± 0.117 U $mg^{-1}$ protein respectively ($P = 0.02$). The cell lines, belonging to the group at the left in Figure 1, were called type I and the cell lines on the right type II.

*NSE-immunoreactivity (NSE-ag), total enolase- and γ-enolase enzyme activity*

As shown in Figure 2 the ratio between NSE-ag content and total enolase activity, measured in the same cell lines of the paragraph above, also distinguished the same two types of cell lines with the exception of GLC 1–13 and 16 (open squares). The mean ratio for type I cell lines was 635 ± 144 ng $U^{-1}$ and for the right group 2994 ± 350 ng $U^{-1}$ ($P<0.0001$). These data indicated the existence of an abrupt increase of NSE-ag without enzyme activity between the two types of cell lines. The ratios for the two exceptional cell lines were 1193 and 1294 respectively. Both cell lines had NSE-ag contents in earlier passages consistent with type II cell lines. Six and nine passages later the Td had decreased from 56 to 35 h and from 60 to 39 h respectively at which time the ratio

**Figure 1** Correlation between intracellular NSE-ag content and Td during well-controlled exponential growth. Correlation coefficients are 0.95 and 0.99 respectively and *P*-valves $<0.0001$. ● ( = variant) plus ■ ( = classic) are called type I, ▲ ( = classic) is called type II.

**Figure 2** Correlation between intracellular NSE-ag and enolase enzyme activity during well-controlled exponential growth. ● ( = variant) plus ■ ( = classic) are called type I, ▲ ( = classic) is called type II, □ = intermediate cell lines between type I and II.

between enolase-activity and NSE-ag content completely fitted in the type I cell lines. This observation suggested that the intermediate values, shown in Figure 2, were due to a mixture of type I and II cell lines on their way to type I cell lines. In order to investigate this hypothesis a type II sample of GLC-16 with a Td of 60 h and a type I sample of GLC-16 with a Td of 28 h were cultured as a 1:1 mixture. At the start of the mixed culture the enolase/NSE-ag ratio showed an intermediate value. At each passage the Td and enolase/NSE-ag ratio decreased until after 5 passages a Td of 30 h and an enolase/NSE-ag ratio of 700 was reached and remained constant thereafter (data not shown). These data support the existence of an abrupt instead of a gradual change of NSE-ag without enzyme activity.

In a separate experiment with 24 different passages of GLC-1, 8, 11, 14, 19, 28 and 34 the percentage of enzymatically active αα, αγ, γγ chains was measured. The percentage of enzymatically active γ-chains was calculated by adding half of the percentage of αγ-isoenzymes and two times the percentage of γγ-isoenzymes. NSE-ag was determined in only 13 of these cell lines. The ratio between γ-enolase activity and NSE-ag level again distinguished two types of cell lines, which were further characterised by a significant difference of the mean enolase activity, mean NSE-ag content and mean Td and an abrupt increase of NSe-ag without enzyme activity between both types (data not shown). In addition the mean γ-enolase activity of the two groups was $33.5 \pm 29$ mU mg$^{-1}$ protein and $150 \pm 45$ mU mg$^{-1}$ protein ($P < 0.0001$) respectively and the mean percentage of enzymatically active γ-chains $4.2 \pm 2.3\%$ and $25.9 \pm 3.6\%$ ($P < 0.0001$) respectively. As shown in Figure 3 a significant correlation was found between the log γ-enolase activity and log NSE-ag content in both types ($y = 1.39 \times - 1.11$, $r = 0.94$, $P = 0.001$ for type I; $y = 1.28 \times - 1.83$, $r = 0.97$, $P = 0.005$ for type II). Interestingly the slope of both regression lines was almost identical. These data indicate that the logarithmic increase of γ-enolase activity is significantly correlated with the logarithmic increase of NSE-ag content in an almost identical way in both types of cell lines but at a different NSE-ag level. The NSE-ag level shows an abrupt increase without enzyme activity between both types.

The percentage of enzymatically active γ-chains in type I cell lines was significantly correlated with the γ-enolase activity. However no such correlation was observed in type II cell lines (Figure 4).

*Immunoblotting*

An immunoblotting assay with polyclonal rabbit antibodies against human γγ-enolase was performed on extracts of a variant (GLC-2), a transitional (GLC-8) and a classic (GLC-34) cell line. Purified human γγ-enolase and human brain cortex grey matter were used as references. All cell lines showed only immunoreactivity migrating on gel as γγ-enolase.

### Discussion

The relevance of the distinction between classic, transitional and variant cells *in vitro* has not been demonstrated *in vivo*. This is mainly due to the heterogeneity of the tumour and difficulty to obtain representative and sufficient tumour material from patients. Therefore our attention focused on tumour markers as a possible source of information about the composition of SCLC-tumours *in vivo*. Neuron-specific enolase (NSE) is one of the most widely used tumour markers in SCLC. It has been shown to be a clinically reliable tool to monitor the course of the disease (Cooper *et al*., 1985; Splinter *et al*., 1987*a*; Splinter *et al*., 1989), the doubling-time of NSE at relapse was highly significantly correlated with survival from time of relapse (Splinter *et al*., 1987*b*) and pretreatment-values were shown to have prognostic value by some (Jørgenson *et al*., 1988), but not by others (Van der Gaast *et al*., 1991). Moreover NSE is a good marker for neuronal differentiation and maturation (Schmechel *et al*., 1980). However the presence of NSE in many non-neuronal and non-neuroendocrine tissues together with a lack of understanding how a relatively unimportant

**Figure 3** Correlation between intracellular log γ-enolase activity (mU mg$^{-1}$ protein) and log NSE (ng mg$^{-1}$ protein). ● ( = variant) plus ■ ( = classic) are called type I, ▲ ( = classic) is called type II.

**Figure 4** Correlation between the percentage of enzymatically active γ-chains and γ-enolase activity. ● ( = variant) plus ■ ( = classic) are called type I and ▲ ( = classic) is called type II.

glycolytic enzyme might play such a distinct role in differentiation raised the question whether NSE was not neuronspecific but nonspecific (Schmechel, 1985). Therefore we started to investigate the relationship between NSE and biological behaviour in SCLC-cell lines. Two types of cell lines could be distinguished by significant differences of NSE-ag content, total enolase- and γ-enolase enzyme activity, Td and an abrupt appearance or increase of NSE-ag without enzyme activity, which was reflected in significantly different NSE-ag/enolase ratios and NSE-ag/γ-enolase ratios. In both types the NSE-ag content and γ-enolase activity (data not shown) were linearly correlated with the Td, albeit at two different levels. It is therefore concluded that γ-enolase activity seems to be associated with the regulation of growth rate. In addition in type II cell lines, but not in type I cell lines, a dissociation was observed between the percentage of enzymatically active γ-chains and γ-enolase activity. This may indicate that in type II cell lines either the production of enzymatically active γ-chains is dissociated from the production of α-chains or active γ-chains are inactivated by a mechanism, which acts independent of the regulation of production. Recently it was shown that V-src could induce phosphorylation of glycolytic enzymes, such as enolase and especially γ-enolase (Cooper et al., 1983). Phosphorylation of γ-enolase led to partly inactivation of the enzyme, accompanied by an increase of the total amount of the enzyme (Eigenbrodt et al., 1983). Moreover c-*src* expression in neuroblastoma- and SCLC-cell lines correlated with neuroendocrine differentiation (Mellström et al., 1987) and c-*src* is connected to neurogenesis and neuronal differentiation (Brickel et al., 1991), as is the switch from α to γ-enolase (Schmechel et al., 1980). In this connection it is very interesting that Wevers et al. (1988) found in cerebrospinal fluid but not in serum from healthy individuals that 50% of NSE-ag had no enzyme activity. These data suggest that NSE-ag without enzyme activity may arise from inactivation of γ-chains and may be correlated with neuronal or neuroendocrine differentiation. However, it is also possible that inactivation of γ-enolase by phosphorylation merely reflects protein kinase activity, leading to different changes in different cell types. With polyclonal rabbit antibodies against γγ-enolase we could not demonstrate the presence of a compound which migrated differently from γγ-enolase. Whatever the explanation, characterisation of NSE-ag without enzyme activity, investigation of the regulation of its production and of the production of active γ-enolase may produce more information about the growth rate and differentiation of SCLC-cell lines.

The data presented in this paper support the distinction of two new classes of SCLC-cell lines, type I and II, with different biological characteristics. Possibly the classic cell lines, belonging to type I, are similar to the transitional cell lines, characterised by the presence of p64 c-*myc* (Bepler et al., 1989*b*). Whether such a distinction *in vitro* has any relevance *in vivo* should be and possibly can be investigated by measuring the ratio between NSE-ag and γ-enolase activity in serum samples from patients with SCLC. A comparison of enzyme activity and immunoreactivity by Sorensen et al. (1988) in serum samples from five SCLC-patients showed that at least in some samples the amount of NSE-ag and γ-enolase activity were significantly different.

Finally, the demonstration that 'neuron-specific enolase' measured with an antibody, can be the enzyme NSE or an enzymatically inactive compound emphasises that further investigations about neuron-specificity of NSE needed. It may be that NSE immunoreactivity in neuroendocrine cells is different from the one in non-neuroendocrine cells.

We are very grateful for the critical comments and suggestions from Prof D. Bootsma and Dr A. de Klein. Moreover we like to thank Dr L. de Ley for providing us with the cell lines.

### References

ARIYOSHI, Y., KATO, K., ISHIGURO, Y., OTA, K., SATO, T. & SUCHI, T. (1983) Evaluation of serum neuron-specific enolase as a tumour marker for carcinoma of the lung. *Gann*, **74**, 219-225.

AURELL, A., ROSENGREN, L.E., NORDBERG, C. & HAGLID, K.G. (1989). The S-100 protein in cerebrospinal fluid: a simple ELISA method. *J. Neurol. Sci.*, **89**, 157-164.

BEPLER, G., JAQUES, G., NEUMANN, K., AUMÜLLER, G., GROPP, C. & HAVERMANN, K. (1987). Establishment, growth properties, and morphological characteristics of permanent human small cell lung cancer cell lines. *J. Cancer Res. Clin. Oncol.*, **113**, 31-40.

BEPLER, G., OSTHOLT, M., NEUMANN, K., HOLLE, R., JAQUES, G., HAVEMANN, K. & KALBFLEISCH, H. (1989*a*). Carcinoembryonic antigen as differentiation marker for small cell lung cancer *in vitro* and its clinical relevance. *Anticancer Res.*, **9**, 1525-1530.

BEPLER, G., BADING, H., HEIMANN, B., KIEFER, P., HAVEMANN, K. & MOELLING, K. (1989*b*). Expression of p64c-myc and neuroendocrine properties define three subclasses of small cell lung cancer. *Oncogene*, **4**, 45-50.

BOOMSMA, F., VAN DER HOORN, F.A.J. & SCHALEKAMP, M.A.D.H. (1986). Determination of aromatic L-amino acid decarboxylase in human plasma. *Clin. Chim. Acta*, **159**, 173-183.

BRICKELL, P.M. (1991). The c-src family of protein-tyrosine kinases. *Int. J. Exp. Pathol.*, **72**, 97-108.

BROERS, J.L.V., CARNEY, D.N., DE LEY, L., VOOIJS, G.P. & RAMAEKERS, F.C.S. (1985). Differential expression of intermediate filament protein distinguishes classic from variant small cell lung cancer cell lines. *Proc. Natl. Acad. Sci,*, **82**, 4409-4413.

BROERS, J.L.V., PAHLPLATZ, M.M., KATZKO, M.W., OUD, P.S., RAMAEKERS, F.C.S., CARNEY, D.N. & VOOIJS, G.P. (1988). Quantitative description of classic and variant small cell lung cancer cell lines by nuclear image cytometry. *Cytometry*, **9**, 426-431.

CARNEY, D.N., GAZDAR, A.F., BEPLER, G., GUCCION, J.G., MARANGOS, P.J., MOODTY, T.W., ZWEIG, M.H. & MINNA, J.D. (1985). Establishment and identification of small cell lung cancer cell lines having classic and variant features. *Cancer Res.*, **45**, 2913-2923.

COOPER, J.A., REISS, N.A., SCHWARTZ, R.J. & HUNTER, T. (1983). Three glycolytic enzymes are phosphorylated at tyrosine in cells transformed by Rous sarcoma virus. *Nature*, **302**, 218-223.

COOPER, E.H., SPLINTER, T.A.W., BROWN, D.A., MUERS, M.F., PEAKE, M.D. & PEARSON, S.L. (1985). Evaluation of a radioimmunoassay for neuron specific enolase in small cell lung cancer. *Br. J. Cancer*, **52**, 333-338.

EIGENBRODT, E., FISTER, P., RÜBSAMEN, H. & FRIIS, R.R. (1983). Influence of transformation by Rous sarcoma virus on the amount, phosphorylation and enzyme kinetic properties of enolase. *EMBO J.*,**2**, 1565-1570.

GAAST, A., VAN DER PUTTEN, W.L.J., VAN OOSTEROM, R., COZIJNSEN, M., HOEKSTRA, R. & SPLINTER, T.A.W. (1991). Prognostic value of serum thymidine kinase, tissue polypeptide antigen and neuron specific enolase in patients with small cell lung cancer. *Br. J. Cancer*, **64**, 369-372.

GAZDAR, A.F., CARNEY, D.N., NAU, M.M. & MINNA, J.D. (1985). Characterisation of variant subclasses of cell lines derived from small cell lung cancer having distinctive biochemical, morphological, and growth properties. *Cancer Res.*, **45**, 2924-2930.

HAGLID, K.G., CARLSSON C.A. & STAVROU, D. (1973). An immunological study of human brain tumours concerning the brain-specific proteins S-100 and 14.3.2. *Acta Neuropath.*, **24**, 187-196.

HULLIN, D.A., BROWN, K., KYNOCH, P.A.M., SMITH, C. & THOMPSON, R.J. (1980). Purification, radioimmunoassay, and distribution of human brain 14-3-2 protein (nervous-system specific enolase) in human tissues. *Biochim. Biophys. Acta*, **628**, 98-108.

JØRGENSON, J.G.M., OSTERLIND, K., HANSEN, H.H. & COOPER, E.H. (1988). The prognostic influence of serum neuron specific enolase in small cell lung cancer. *Br. J. Cancer*, **58**, 805-807.

KUZMITS, R., SCHERNTHANER, G. & KRISCH, K. (1987). Serum neuron-specific enolase, a marker for response to therapy in seminoma. *Cancer*, **60**, 1017-1021.

LEIJ, DE, L., POSTMUS, P.E., BUYS, C.H.C.M., ELEMA, J.D., RAMAEKERS, F., POPPEMA, S., BROUWER, M., VAN DER VEEN, A.Y., MESANDER, G. & THE, T.H. (1985). Characterization of three new variant type cell lines derived from small cell carcinoma of the lung. *Cancer Res.*, **45**, 6024–6033.

MARANGOS, P.J., CAMPBELL, I.C., SCHMECHEL, D.E., MURPHY, D.L. & GOODWIN, F.K. (1980*b*) Blood platelets contain a neuron-specific enolase subunit. *J. Neurochem.*, **34**, 1254–1258.

MELLSTRÖM, K., BJELFMAN, C., HAMMERLING, U. & PÅHLMAN, S. (1987). Expression of c-src in cultured human neuroblastoma and small cell lung carcinoma cell lines correlates with neurocrine differentiation. *Mol. & Cell. Biol.*, **7**, 4178–4184.

MOODY, T.W., CARNEY, D.N., KORMAN, L.Y., GAZDAR, A.F. & MINNA, J.D. (1985). Neurotensin is produced by and secreted from classic small cell lung cancer cells. *Life Sci.*, **36**, 1727–1732.

NIEHANS, G.A., MANIVEL, J.C., COPLAND, G.T., SCHEITHAUER, B.W. & WICK, M.R. (1988). Immunohistochemistry of germ cell and trophoblastic neoplasms. *Cancer*, **62**, 1113–1123.

OKA, K., MORI, N., HAIMOTO, H. & KATO, K. (1989). Expression of enolase in B cell tumours. *Lab. Invest.*, **60**, 38–44.

OSKAM, R., RIJKSEN, G., LIPS, C.J.M. & STAAL, G.E.J. (1985). Enolase Isoenzymes in differentiated and undifferentiated medullary thyroid carcinomas. *Cancer*, **55**, 394–399.

PINTO, A., GRANT, L.H., HAYES, F.A., SCHELL, M.J. & PARHAM, D.M. (1989). Immunohistochemical expression of neuron-specific enolase and leu 7 in Ewing's sarcoma of bone. *Cancer*, **64**, 1266–1273.

SCHMECHEL, D., MARANGOS, P.J. & BRIGHTMAN, M. (1978). Neuron-specific enolase is a molecular marker for peripheral and central neuroendocrine cells. *Nature*, **276**, 834–836.

SCHMECHEL, D.E., BRIGHTMAN, M.W. & MARANGOS, P.J. (1980). Neurons switch from non-neuronal (NNE) enolase to neuronal (NSE) enolase during development. *Brain Res.*, **190**, 195–214.

SCHMECHEL, D.E. (1985). Editorial. γ-Subunit of the glycolytic enzyme enolase: nonspecific or neuron specific? *Lab. Invest.*, **52**, 239–242.

SORENSEN, K., BRODBECK, U., PAUS, E. & NORGAARD-PEDERSEN, B. (1988). An enzyme antigen immunoassay for the determination of neuron-specific enolase in serum samples. *Clin. Chim. Acta*, **175**, 337–344.

SPLINTER, T.A.W., COOPER, E.H., KHO, G.S., OOSTEROM, R. & PEAKE, M.D. (1987*a*). Neuron specific enolase as a guide to the treatment of small cell lung cancer. *Eur. J. Cancer Clin. Oncol.*, **23**, 171–176.

SPLINTER, T.A.W., COOPER, E.H. & OOSTEROM, R. (1987*b*). Doubling-time of neuron specific enolase and survival in small cell lung cancer patients, result of a preliminary analysis. *Eur. J. Resp. Dis.*, Suppl. **149**, Vol 70; 37–44.

SPLINTER, T.A.W., CARNEY, D.N., TEELING, M., PEAKE, M.D., KHO, G.S., OOSTEROM, R. & COOPER, E.H. (1989). Neuron specific enolase as a sole guide to treat small cell lung cancer patients in common clinical practice. *J. Cancer Res. Clin. Oncol.*, **115**, 400–401.

TAKASHI, M., HAIMOTO, H., TANAKA, J., MURASE, T. & KATO, K. (1989). Evaluation of gamma-enolase as a tumour marker for renal cell carcinoma. *J. Urol.*, **141**, 830–834.

TAPIA, F.J., POLAK, J.M., BARBOSA, A.J.A., BLOOM, S.R., MARANGOS, P.J. & DERMODY, C. (1981). Neuron specific enolase is produced by neuroendocrine tumours. *Lancet*, **1**, 808–812.

TOWBIN, H., STAEHELIN, T. & GORDON, J. (1979). Electrophoretic transfer of proteins from polyacrylamide gels to nitrocellulose sheets: procedure and some applications. *Proc. Natl. Acad. Sci. USA*, **76**, 4350–4354.

WANG, S., ROSENGREN, L.E., KARLSSON, J.E., STIGBRAND, T. & HAGLID, K.G. (1990). A simple quantitative dot-immunobinding assay for glial and neuronal marker protein in SDS-solubilized brain tissue extracts. *J. Neurosci. Methods*, **33**, 219–227.

WEVERS, R.A., THEUNISSE, A.W.G. & RIJKSEN, G. (1988). An immunobioluminescence assay for gamma-gamma enolase activity in human serum and cerebrospinal fluid. *Clin. Chim. Acta*, **178**, 141–150.

WICK, M.R., BERND, M.D., SCHEITHAUER, W. & KOVACS, K. (1983). Neuron specific enolase in neuroendocrine tumours of the thymus, bronchia and skin. *Am. J. Clin. Pathol.*, **79**, 703–707.